# Association between early radiographic chest findings and clinical outcomes in pediatric drowning: a retrospective study in a tertiary Italian hospital

**DOI:** 10.1007/s00431-025-06029-4

**Published:** 2025-02-11

**Authors:** Tommaso Bellini, Giacomo Brisca, Matteo D’Alessandro, Jessica Tibaldi, Valentina Andreottola, Chiara Conti, Federica Casabona, Elisabetta Lampugnani, Emanuela Piccotti, Andrea Moscatelli

**Affiliations:** 1 Paediatric Emergency Room and Emergency Medicine Unit, IRCCS Istituto Giannina Gaslini, Via G. Gaslini, 5, 16147 Genoa, Italy; 2 Paediatric and Neonatal Intensive Care Unit, IRCCS Istituto Giannina Gaslini, Genoa, Italy; 3 Department of Neuroscience, Rehabilitation, Ophthalmology, Genetics, Maternal and Child Health (DINOGMI), University of Genoa, Genoa, Italy

**Keywords:** Chest imaging, Drowning, Pediatric emergency department, Respiratory support, Prognosis

## Abstract

Pediatric drowning incidents, both fatal and non-fatal, represent a significant challenge in emergency medicine, particularly for children under 14 years of age. Drowning is a leading cause of unintentional injury-related deaths, with children aged one to four years being especially vulnerable. Accurate and timely assessment, particularly through chest imaging, such as chest radiography (CXR) and lung point-of-care ultrasound (LUS), is crucial for evaluating pulmonary complications and guiding treatment decisions. This retrospective study analyzed 64 pediatric drowning cases in Istituto Giannina Gaslini, Italy, from 2012 to 2023. For all patients, demographic data, vital parameters, blood tests upon PED admission and at 24 h, need for intensive care unit admission, length of stay, therapies administered, ventilatory support in the PED and in the hospital ward, and mortality were recorded. The patients were subsequently divided into two groups based on the chest X-ray result. The results indicated that pathological CXR findings upon admission were associated with a higher likelihood of hospital admission, prolonged hospital stay, and more severe clinical outcomes, including the need for oxygen supplementation and antibiotic therapy. Patients with abnormal CXR findings also exhibited lower oxygen saturation, Glasgow Coma Scale scores, and body temperature upon admission, reflecting a more significant physiological compromise, and higher RCP values at 24 h.

*Conclusion*: Although the majority of non-fatal drowning patients recovered without lasting neurological deficits, the findings suggest that CXR plays a critical role in early management, assisting in the identification of those at greater risk for complications.

**Table:** 

**What is Known:** *• Fatal and non-fatal pediatric drowning incidents may represent a critical issue in pediatric emergency medicine, requiring prompt assessment and accurate management upon admission, even if standardized protocols are lacking.*
**What is New:** *• Chest imaging in the initial assessment of pediatric drowning cases may serve as a fundamental tool to support early clinical decision-making, particularly when integrated with other key parameters such as neurological status, vital signs, and blood exams.*

**What is Known:**

*• Fatal and non-fatal pediatric drowning incidents may represent a critical issue in pediatric emergency medicine, requiring prompt assessment and accurate management upon admission, even if standardized protocols are lacking.*

**What is New:**

*• Chest imaging in the initial assessment of pediatric drowning cases may serve as a fundamental tool to support early clinical decision-making, particularly when integrated with other key parameters such as neurological status, vital signs, and blood exams.*

## Introduction

Fatal and non-fatal pediatric drowning incidents represent a critical challenge in emergency medicine, especially among children up to 14 years of age, requiring prompt assessment and accurate management upon admission [1–5].

According to the World Health Organization (WHO), drowning ranks as the third leading cause of unintentional injury-related death worldwide, and in children aged one to four years it stands as the primary preventable cause of mortality, particularly in low and middle-income countries [1, 4–7].

Timely and adequate assessment of these cases is imperative as it facilitates the initiation of appropriate interventions and the improvement of patient outcomes. While chest imaging is widely used in clinical practice, its precise role in pediatric drowning cases remains still, partially underexplored [2, 4]. Current literature lacks robust evidence on the prognostic utility of chest imaging in pediatric drowning cases, for stratifying severity and guiding clinical decisions in the early hours after Pediatric Emergency Department (PED) admission. Among the available diagnostic tools for chest imaging in a PED, chest radiography (CXR) and, more recently, lung point-of-care ultrasound (LUS), may play a crucial role in evaluating pulmonary complications associated with submersion events in pediatric patients [1–4].

While existing studies focus on general drowning management, our study aims to provide evidence for a more targeted approach, specifically assessing the role that CXR findings upon PED admission for fatal and non-fatal pediatric drowning episodes may have on risk stratification, clinical decision-making, and patient outcomes.

## Material and methods

### Study setting and data collection

This single-center retrospective study included all children aged 0–14 admitted for drowning from January 1, 2012, to December 31, 2023, to the PED of IRCCS Istituto Giannina Gaslini, Genoa, Italy. Istituto Giannina Gaslini is a tertiary care children’s hospital serving the Liguria region in northwestern Italy, receiving an average of 37,000 PED visits per year from patients aged 0–14 years. All retrospective data were collected from electronic medical records.

For all patients, demographic data (age, sex, and comorbidities), severity of events in terms of associated witnessed head or spine trauma, loss of consciousness (LOC), need for cardiopulmonary resuscitation (CPR) and intubation at the scene, vital parameters, blood tests and CXR upon PED admission and at 24 h, need for intensive care unit (ICU) admission, length of stay, therapies administered, ventilatory support in the PED and in the hospital ward, and mortality were recorded. CPR was defined as ventilatory assistance and/or chest compression. The chart review process adhered to the methodological standards described by Gilbert et al., ensuring consistency and reliability in data extraction [8]. We followed a structured protocol for reviewing medical records, focusing on the predefined variables reported above [8].

### Definition of vital parameters and chest imaging

Normal age-based respiratory rate, heart rate, and blood pressure values were adopted from the Pediatric Advanced Life Support (PALS) criteria [8]. Body temperature was considered normal between 36 and 37.5 °C. Pulse oximetry and heart rate were continuously monitored using transcutaneous probes placed over a single digit.

Respiratory distress included any signs of augmented work of breathing. Normal mentation was defined as a pediatric Glasgow Coma Score (pGCS) of 15 [2, 9].

Patients were subsequently categorized into two groups based on the CXR report and all collected data were correlated with the CXR findings. It is noteworthy that no standardized severity scale exists for grading CXR, and the literature on CXR findings associated with drowning is limited [4]. Consequently, CXRs were classified as normal if there was no evidence of pulmonary involvement or abnormal if the final report indicated atelectasis, edema, infiltrates, opacities, or effusions [2, 4].

CXRs were obtained upon PED admission for all patients within 90 min of their arrival. Radiographs were taken either as anteroposterior views at the bedside or as standard radiographic images in the PED radiology suite, depending on the patient’s clinical condition and stability. When the patient was unstable or required urgent resuscitation, bedside imaging was performed to minimize any delays in care. All CXR images were interpreted by pediatric radiologists on duty with at least 5 years of experience in pediatric imaging. The radiologists provided a final report, which was used to classify CXR findings as either normal or abnormal.

### Statistical analysis

Kruskal–Wallis test or Mann–Whitney *U*-test for continuous variables and chi-square or Fisher’s exact test for categorical variables were used to access differences between the groups. Statistical significance was set at *p* < 0.05, and all values were determined using two-tailed tests. All statistical analyses were conducted using IBM SPSS Statistics for Windows Version 21.0 (IBM Corp. Armonk, NY, USA).

## Results

A total of 64 patients under 14 years of age were enrolled, with an annual median of five patients per year, resulting in an overall incidence of 1.5/10,000 PED admissions. Two patients did not undergo CXR and were consequently excluded. The key data are summarized in Tables 1, 2, and 3. Only one patient (1.5%) died. Consequently, in the text, the identification of patients as survivors or non-survivors of the drowning incident has been omitted, and the term “drowning” has been applied uniformly to both the deceased patient and the remaining surviving patients. The median age of the patients was 5.5 (3.3–8.6) years, with 64% being male, and there was no statistically significant difference between the two groups. All drowning events occurred during the spring–summer months (May–October), predominantly in saltwater environments. Among comorbidities, two patients had developmental delay, one had developmental delay and low vision, two patients had asthma, one patient had spinal muscular atrophy type 2, and one patient was on antiepileptic drugs for rolandic epilepsy. No other patients had chronic lung disease.

**Table 1:** Demographic and clinical characteristics of patients

	Total *n* = 64	Normal Chest X-ray *n* = 29	Pathological Chest X-ray *n* = 35	*p*
Sex, *male (%)*	41 (64%)	17 (58.5%)	24 (68.5%)	0.40
Age in years, *median (IQR 25–75)*	5.5 (3.4–8.6)	6.4 (4.2–8.8)	4.5 (3.0–8.5)	0.56
Saltwater, *yes (%)*	45 (70%)	18 (62%)	27 (77%)	0.18
Comorbidities, *yes (%)*	8 (12.5%)	3 (4.5%)	5 (14%)	0.60
LOC, *yes (%)*	33 (51.5%)	16 (66.5%)	17 (48.5%)	0.59
CPR, *yes (%)*	20 (31%)	10 (34.5%)	10 (28.5%)	0.61
Trauma, *yes (%)*	9 (14%)	2 (7%)	7 (20%)	0.13
Body temperature in °C, *median (IQR 25–75)*	36.3 (36.0–37.0)	36.2 (36.0–36.6)	36.5 (36.0–37.5)	0.18
Hypothermia,* yes (%)*	8 (33%)	1 (3.5%)	7 (20%)	**0.042**
SaO_**2**_ (%), *median (IQR 25–75)*	93 (88–98)	97 (93–99)	91 (87–94)	**0.0065**
SaO_**2**_ < 95%, *yes (%)*	34 (53%)	10 (34.5%)	24 (68.5%)	**0.0035**
Respiratory rate (per minute), *median (IQR 25–75)*	28 (24–36)	24 (20–32)	32 (24–40)	**0.0035**
Tachydyspnea, *yes (%)*	33 (51.5%)	9 (31%)	24 (68.5%)	**0.0028**
Pulse rate (per minute), *median (IQR 25–75)*	120 (110–138)	118 (106–127)	122 (115–147)	0.091
Bradycardia, *yes (%)*	4 (6%)	0 (0%)	4 (11.5%)	0.10
pGCS, *median (IQR 25–75)*	15 (14–15)	15 (15–15)	15 (14–15)	0.12
pGCS ≤ 14, *yes (%)*	19 (29.5%)	5 (17%)	14 (40%)	**0.047**
Systolic BP in mmHg, *median (IQR 25–75)*	103 (98–111)	105 (99–110)	101 (96–115)	0.66
Diastolic BP in mmHg, *median (IQR 25–75)*	62 (52–69)	64 (55–69)	60 (52–68)	0.67
Hypotension,* yes (%)*	7 (11%)	1 (3.5%)	6 (17%)	*0.08*
Admission, *yes (%)*	60 (93.5%)	25 (86%)	35 (100%)	**0.023**
ICU admission, *yes (%)*	2 (3%)	1 (3.5%)	1 (3%)	0.34
Death, *yes (%)*	1 (1.5%)	0 (0%)	1 (3%)	0.35
Length of stay in days, *median (IQR 25–75)*	3 (3–4)	3 (2–3)	4 (3–5)	**0.046**

*LOC*, loss of consciousness; *CPR*, cardiopulmonary resuscitation; *SaO*_***2***_, transcutaneous arterial capillary oxygen saturation; *GCS*, pediatric Glasgow Coma Scale; *BP*, blood pressure; *ICU*, Intensive Care Unit; *IQR*, interquartile range

**Table 2:** Blood test results

	Total *n* = 64	Normal Chest X-ray *n* = 29	Pathological Chest X-ray *n* = 35	*p*
pH, *median (IQR 25–75)*	7.30 (7.26–7.34)	7.32 (7.28–7.34)	7.30 (7.25–7.35)	0.96
HCO_**3**_ in mEq/l, *median (IQR 25–75)*	18.0 (16.4–20.6)	19.0 (17.0–20.0)	18.0 (16.0–21.0)	*0.08*
pCO_**2**_ in mmHg, *median (IQR 25–75)*	47.0 (41.0–51.0)	48.0 (41.0–50.0)	47.0 (41.0–53.0)	0.84
Lactate in mg/dl, *median (IQR 25–75)*	15.0 (11.0–21.3)	14.0 (11.0–21.0)	15.5 (11.0–21.0)	0.96
Na^**+**^ in mEq/l, *median (IQR 25–75)*	143 (138–149)	141 (134–149)	144 (139–149)	0.8
Cl^**−**^ in mEq/l, *median (IQR 25–75)*	108 (104–115)	107 (101–113)	109 (106–116)	0.64
CRP *t*^***0***^ in mg/dl, *median (IQR 25–75)*	0.00 (0.00–0.00)	0.00 (0.00–0.00)	0.00 (0.00–0.00)	0.54
CRP *t*^***24***^ in mg/dl, *median (IQR 25–75)*	1.24 (0.00–4.21)	0.38 (0.00–0.93)	2.03 (0.76–5.70)	***0.01***
WBC *t*^***0***^ in cells/mm^3^, *median (IQR 25–75)*	12,800 (11,120–15,120)	13,000 (9530–14,000)	12,600 (11,750–16,320)	0.7
WBC *t*^***24***^ in cells/mm^3^, *median (IQR 25–75)*	9980 (8080–13,585)	9490 (5850–11,650)	11,360 (8,380–14,000)	0.54

*HCO*_***3***_^***−***^, serum bicarbonates; *pCO*_***2***_, venous partial pressure CO_2_; *Na*^**+**^, natriemia; *Cl*^***−***^, chloremia; *CRP*, C-reactive protein; *WBC*, white blood cells; *IQR*, interquartile range

**Table 3:** Therapies administered at PED admission and in hospital ward

	Total *n* = 64	Normal Chest X-ray *n* = 29	Pathological Chest X-ray *n* = 35	*p*
O_**2**_ supplementation, *yes (%)*	46 (72%)	14 (48%)	32 (91.5%)	***0.0001***
Steroids, *yes (%)*	23 (36%)	8 (27.5%)	15 (43%)	0.2
Antibiotics, *yes (%)*	52 (81%)	18 (62%)	34 (97%)	***0.0003***
Fluid resuscitation, *yes (%)*	8 (12.5%)	3 (10.5%)	5 (14%)	0.69
HFNC/CPAP, *yes (%)*	13 (20%)	5 (17%)	8 (23%)	0.57
Length of O_**2**_ supplementation in hours *median (IQR 25–75)*	6 (3–12)	9 (3–12)	6 (3–12)	0.98
Length of HFNC/CPAP in hours *median (IQR 25–75)*	24 (18–24)	18 (12–24)	24 (24–30)	0.56

*HFNC*, high-flow nasal cannula; *CPAP*, continuous positive airway pressure; *IQR*, interquartile rang

Patients with pathological CXR findings on admission exhibited a significantly higher risk of hospital admission (*p* = 0.023). Moreover, these patients demonstrated a propensity for extended hospital stays (*p* = 0.046). Nevertheless, no statistically significant differences were observed between the two groups regarding LOC, necessity for CPR, associated trauma, or ICU admission.

Patients with abnormal CXR findings on admission exhibited significantly higher respiratory rates (*p* = 0.0035), lower oxygen saturation levels (*p* = 0.0065), reduced pGCS scores (*p* = 0.047), and lower body temperatures (*p* = 0.042).

Blood tests on admission were comparable between the two groups. However, patients with abnormal CXR findings demonstrated significantly higher C-reactive protein (CRP) levels at 24 h post-admission (*p* = 0.02).

Patients with abnormal CXR findings were more likely to require greater intervention, including oxygen supplementation (*p* = 0.0001) and parenteral antibiotic therapy (*p* = 0.003). Only one patient died in the pathological CXR group.

## Discussion

Drowning is defined as respiratory impairment resulting from submersion or immersion in any liquid, with outcomes ranging from death to non-fatal injury or no injury [1, 6]. This definition emphasizes the primary role of acute respiratory failure in the pathophysiology. Clinical manifestations are predominantly determined by the volume of water aspirated and its deleterious effects on the alveolar-capillary membrane, leading to bronchospasm, loss of surfactant, increased alveolar-capillary membrane permeability, ventilation/perfusion mismatch, atelectasis, aspiration pneumonia, and acute respiratory distress syndrome (ARDS) [1, 2, 6, 10–12]. These conditions may potentially result in hypercapnia, hypoxemia, and secondary cardiac arrest, subsequently leading to neurologic morbidities, which constitute the most significant prognostic factors for predicting long-term outcomes [6, 11]. It is noteworthy that in the majority of patients successfully rescued from drowning, circulatory function is restored following prompt oxygenation, crystalloid infusion, and normalization of body temperature, thus confirming that timely rescue and management are the most crucial prognostic factors [1, 12]. Our findings suggest that lower SaO2 upon admission to the PED may serve as a valid indicator of clinical severity and lung involvement. This observation confirms that oxygen administration is a fundamental component in the management of drowning patients and should be initiated without delay [4, 8, 12]. Furthermore, we observed that patients presenting with pathological CRX exhibited lower pGCS and reduced body temperatures, reflecting the severity of their circulatory and neurological compromise during the drowning incident [6]. Insufficient data precludes definitive conclusions regarding optimal respiratory support for pediatric drowning victims, and the literature lacks substantial evidence to establish a standard protocol [10]. However, in our investigation, the utilization of high-flow nasal cannula (HFNC) and/or continuous positive airway pressure (CPAP) did not demonstrate significant differences between the two cohorts. Consequently, we can only advocate for the application of clinical judgment when determining therapeutic interventions in cases where supplemental oxygen alone proves inadequate [10].

The majority of existing literature on drowning has predominantly focused on adult patients presenting with severe symptomatology necessitating critical care, resulting in a paucity of data addressing the management of well-appearing, mildly symptomatic drowning victims [6, 12, 13]. Historically, it has been suggested that all drowning patients require hospital admission or a period of observation in the emergency department due to the concern that an initially stable patient may develop respiratory distress, hypoxemia, or ARDS several hours or days after the initial event [9, 13, 14].

However, the majority of victims of non-fatal drowning fully recover without neurologic deficits, and recent studies have indicated that pediatric drowning patients who present as clinically stable with normal age-adjusted vital signs upon PED admission can be safely discharged without an extended observation period [1, 2, 13, 14]. In our study, four patients from the negative CXR group were discharged. Moreover, patients in this group exhibited a significantly shorter LOS (*p* = 0.046), indicating that a brief observation period may be sufficient.

Furthermore, our results corroborate that pediatric patients presenting with respiratory distress or abnormal age-appropriate vital signs at PED presentation have a higher likelihood of abnormal initial CRX [2, 15].

Nevertheless, the question of whether any pathological CXR should be considered indicative of pneumonia remains a subject of debate, as initial misdiagnosis may occur due to the early radiographic manifestation of pulmonary edema or atelectasis, which may resolve within 24 h [2]. Consequently, not all initial pathological X-rays should necessitate immediate antibiotic treatment, and it may be advisable to consider hospitalization for all patients with a pathological CXR, to monitor daily fever, inflammatory markers, and the persistence or appearance of new pulmonary infiltrates that may improve or worsen within 24–48 h [1–3, 9, 16]. Therefore, the necessity of antibiotics upon admission remains a subject of debate, but clinicians should be aware of early-onset pneumonia resulting from the aspiration of contaminated water, sand, endogenous flora, or gastric contents [1, 7, 16, 17]. Similarly, there is insufficient evidence to support the prophylactic use of steroids [10].

Notably, our data indicated that patients presenting with an initial pathological CXR exhibited elevated inflammatory markers 24 h post-drowning event, suggesting an intensified inflammatory response and the potential for secondary infections in these individuals, who warrant more intensive and longer clinical monitoring. Interestingly, we did not find any correlation between blood gas measurements and pathological CXR according to previous reports, indicating that blood gas values were only useful in guiding resuscitation or in patients requiring non-invasive ventilation support [6, 10].

Therefore, we reiterate the critical importance of chest imaging in the initial management of drowning survivors.

In this context, LUS may serve as a highly valuable tool for the PED physician, enabling close monitoring of the patient’s lung involvement, providing a more dynamic daily assessment, and potentially reducing the need for additional ionizing radiation or unnecessary antibiotic therapy [3, 17]. LUS demonstrates potentially greater sensitivity than X-ray imaging and represents a readily acquirable skill for fellows, underscoring its pivotal role in the PEDs [18]. Furthermore, LUS can be implemented in prehospital settings, facilitating and informing the management of pediatric drowning victims before transportation to the PED. Finally, its application may resolve a common clinical dilemma regarding the necessity of CRX in apparently asymptomatic pediatric submersion cases [2, 7, 14].

Although our study demonstrated that abnormal CXR findings were associated with SaO2 < 95%, prolonged hospitalization, oxygen supplementation, and increased inflammatory markers, no significant association was found between pathological CXR and ICU admission or the need for more intensive respiratory support. This may be explained by the fact that while CXR abnormalities reflect pulmonary involvement, they do not always correlate with the need for intensive care, which is also influenced by other factors such as the patient’s overall clinical stability, neurological status, associated trauma, response to initial treatments, and CPR on the scene. Thus, further studies are needed to investigate how CXR findings might better predict the need for advanced respiratory support in pediatric drowning victims.

In conclusion, our study demonstrates that CXR abnormalities may be a useful tool to guide physicians in earlier initiation of antibiotics, oxygen supplementation, and closer monitoring in pediatric drowning cases. Conversely, normal CXR findings might support shorter observation periods and earlier discharge in patients who presented clinically stable and alert upon PED admission [2, 6, 7]. The current standard of care, based on previous studies, recommends a 4–8-h observation period, as more than 98% of pediatric patients who developed symptoms did so within the first 4.5 h after the drowning incident [2, 4, 6, 7, 12–14]. Our study contributes to the understanding of how CXR may help guide early clinical decision-making regarding hospitalization and safe discharge.

## Limits and strengths

Our study has several limits. Firstly, the retrospective design limits our ability to establish causal relationships between CXR findings and clinical outcomes. The decision to perform a CXR was clinician-dependent, potentially overrepresenting more severe cases and clinicians might have been influenced by radiographic abnormalities when prescribing antibiotics or determining length of stay. Thus, the associations we observed may be confounded by variables not accounted for in the study. Secondly, we acknowledge the potential for diagnostic bias. Clinicians might have been more likely to prescribe antibiotics or extend hospital stays based on the presence of radiographic abnormalities, independent of the patient’s clinical presentation. However, we believe that this reflects clinical practice, where imaging findings often guide therapeutic decisions. Additionally, we do not know the duration of submersion in most patients, which is universally recognized as the most important prognostic factor. Thirdly, our study was conducted in a single tertiary care center, which may limit the generalizability of our findings to other healthcare settings or patient populations. Moreover, our data are predominantly extrapolated from warm saltwater drowning, which is associated with more favourable outcomes [4, 5, 14]. Finally, while our analysis provides valuable insights into the prognostic role of CXR, we did not assess the inter-observer variability in radiographic interpretation, which could influence the reproducibility of our findings.

Notwithstanding these limitations, our investigation exhibited several strengths. Our study was designed to elucidate the specific role of CXR in pediatric patients who experienced a submersion event. Furthermore, this represents the first Mediterranean case series to address this topic, which may inform practice in other countries with comparable climatic and geographical characteristics.

## Conclusion

These findings highlight the value of chest imaging in the initial assessment of pulmonary involvement in pediatric drowning cases presenting to the PED. CXR can serve as a fundamental tool to support early clinical decision-making, particularly when integrated with other key parameters such as neurological status, vital signs, and blood exams. While abnormal CXR findings may be associated with a clinical profile characterized by compromised physiological parameters and could indicate the need for closer monitoring and intervention, they should not be used in isolation. Similarly, normal lung imaging may support a more favorable prognosis but requires confirmation through comprehensive clinical evaluation. These insights support the incorporation of CXR (or LUS) as part of a multidisciplinary approach to guide management strategies in pediatric drowning scenarios. Future research should explore the development of standardized protocols for their integration into clinical workflows, ensuring efficient resource utilization and optimal patient outcomes.

## Data Availability

The datasets generated and/or analyzed during the current study are available from the corresponding author upon reasonable request.

## Abbreviations

ARDS: Acute respiratory distress syndrome
CPAP: Continuous positive airway pressure
CPR: Cardiopulmonary resuscitation
CRP: C-reactive protein
CXR: Chest X-ray
HFNC: High-flow nasal cannula
ICU: Intensive care unit
LOC: Loss of consciousness
LUS: Lung ultrasound
PALS: Pediatric advanced life support
PED: Pediatric emergency department
pGCS: Pediatric Glasgow Coma Scale
WHO: World Health Organization
